# Breastfeeding in primiparous women with congenital heart disease − a register study

**DOI:** 10.1186/s13006-024-00627-y

**Published:** 2024-03-20

**Authors:** Ylva Holstad, Bengt Johansson, Maria Lindqvist, Agneta Westergren, Inger Sundström Poromaa, Christina Christersson, Mikael Dellborg, Aleksandra Trzebiatowska-Krzynska, Peder Sörensson, Ulf Thilén, Anna-Karin Wikström, Annika Bay

**Affiliations:** 1 Department of Nursing, Umeå University, Umeå, Sweden; 2 Department of Surgical and Perioperative Sciences, Umeå University, Umeå, Sweden; 3 Department of Clinical Sciences, Obstetrics and Gynecology, Umeå University, Umeå, Sweden; 4 Department of Women’s and Children’s Health, Uppsala University, Uppsala, Sweden; 5 Department of Medical Science, Cardiology, Uppsala University, Uppsala, Sweden; 6 Department of Clinical and Molecular Medicine, Sahlgrenska Academy, University of Gothenburg, Gothenburg, Sweden; 7 Department of Cardiology, Department of Medicine and Health Sciences, Linköping University, Linköping, Sweden; 8 Department of Medicine, Solna, Department of Cardiology, Karolinska Institutet, Karolinska University Hospital, Stockholm, Sweden; 9 Department of Clinical Sciences, Cardiology, Lund University, Lund, Sweden

**Keywords:** Heart defects congenital, Breastfeeding, Maternal health, Postpartum period, adult congenital heart disease (ACHD)

## Abstract

**Background:**

The number of pregnant women with congenital heart disease (CHD) is rising, and the disease poses increased risks of cardiovascular and obstetric complications during pregnancy, potentially impacting breastfeeding success. This study aimed to investigate breastfeeding in primiparous women with CHD compared to primiparous women without CHD, and to examine potential hindering factors for breastfeeding in women with CHD.

**Methods:**

The data were gathered between 2014 and 2019 and obtained by merging the Swedish Congenital Heart Disease Register (SWEDCON) with the Swedish Pregnancy Register. Primiparous women ≥ 18 years of age with CHD (*n* = 578) were matched by age and municipality to 3049 women without CHD, giving birth after 22 gestational weeks. Multivariable logistic regression analysis was used to identify factors associated with non-breastfeeding in women with CHD.

**Results:**

Fewer women with CHD breastfed than women without CHD two days (94% vs. 97%, *p* = 0.001) and four weeks after birth (84% vs. 89%, *p* = 0.006). When all women were analysed, having CHD was associated with non-breastfeeding at both two days and four weeks after birth. For women with CHD, body mass index (BMI) ≥ 30 (OR 3.1; 95% CI 1.4, 7.3), preterm birth (OR 6.4; 95% CI 2.1, 19.0), self-reported history of psychiatric illness (OR 2.4; 95% CI 1.2, 5.1), small for gestational age (OR 4.2; 95% CI 1.4, 12.2), and New York Heart Association Stages of Heart Failure class II − III (OR 6.0; 95% CI 1.4, 26.7) were associated with non-breastfeeding two days after birth. Four weeks after birth, factors associated with non-breastfeeding were BMI ≥ 30 (OR 4.3; 95% CI 2.1, 9.0), self-reported history of psychiatric illness (OR 2.2; 95% CI 1.2, 4.2), and preterm birth (OR 8.9; 95% CI 2.8, 27.9).

**Conclusions:**

The study shows that most women with CHD breastfeed, however, at a slightly lower proportion compared to women without CHD. In addition, factors related to the heart disease were not associated with non-breastfeeding four weeks after birth. Since preterm birth, BMI ≥ 30, and psychiatric illness are associated with non-breastfeeding, healthcare professionals should provide greater support to women with CHD having these conditions.

## Background

Breastfeeding is shown to be the most beneficial feeding method during the baby’s first six months of life, and has positive short and long-term health benefits for both mother and baby [1–7]. The World Health Organization (WHO) primarily recommends exclusive breastfeeding due to greater health effects than partial breastfeeding [1, 2]. However, there are advantages to both exclusive and partial breastfeeding when compared to not breastfeeding at all [8, 9]. In Sweden, > 90% of women breastfeed their newborn during the initial days after birth, and > 80% of all two-month-old babies are breastfed: a high level when compared to other European countries [10].

Congenital heart disease (CHD) is the most common birth malformation, seen in 1 / 100 newborns [11]. Thanks to advances in cardiac care, in high-income countries today > 90% of these children reach adulthood [12, 13], which in recent decades, has led to an increase in women with CHD becoming pregnant [14]. However, undergoing pregnancy is not without risks for women with CHD. Cardiovascular events such as heart failure, arrhythmia, and thromboembolism are more common in this group than among healthy women, and obstetric complications such as Caesarean section and preterm birth, known to be breastfeeding-reducing factors, also occur more often for women with CHD [15–17]. Among women in general, both emotional distress and impacts from the environment affect whether a woman continues to breastfeed [17, 18]. In the case of preterm birth, which is emotionally stressful for parents [19], length of stay in intensive neonatal care units has been linked to reduced breastfeeding rates six months after birth for both full-term and pre-term infants [20].

To our knowledge, only one study that previously has described breastfeeding in women with CHD, showed that cardiovascular events did not occur more often among breastfeeding women than among non-breastfeeding women [21]. However, no previous study has evaluated risk factors for non-breastfeeding in women with CHD compared to women without CHD. Data on breastfeeding rates, health, sociodemographic factors, obstetric complications, and factors related to heart disease, can provide healthcare professionals with new knowledge of how to support breastfeeding for women with CHD. This register study therefore, examined breastfeeding rates in women with CHD compared to women without CHD as well as factors that may hinder breastfeeding for women with CHD.

## Methods

### Study design and data source

This register study is based on data covering 2014 − 2019, collected from the Swedish Congenital Heart Disease Register (SWEDCON) and the Swedish Pregnancy Register. SWEDCON is a Swedish national register comprising information on individuals with CHD from diagnosis to end of life. It includes data on aspects such as sociodemographics, diagnoses, interventions, echocardiograms, medications, symptoms linked to heart disease, and New York Heart Association Stages of Heart Failure class (NYHA class) [22]. The Swedish Pregnancy Register is a national register collecting data on pregnancy, childbirth, the postpartum period, reproductive health, and sociodemographic characteristics from > 98% of pregnant women in 16 of the 20 healthcare regions in Sweden. Pregnant women on their first visit to antenatal care, are asked by the midwife about participation in the Swedish Pregnancy Register. Data on health history and diagnoses are downloaded directly from the healthcare region’s electronic medical record into the Swedish Pregnancy Register. Midwives at the maternity ward gather information on breastfeeding rates two days after birth. At the *postpartum* visit, which usually takes place between 6 − 18 weeks *postpartum*, the antenatal care midwife asks retrospectively whether the infant was breastfed at four weeks of age [23].

### Participants and inclusion criteria

**Fig. 1:**
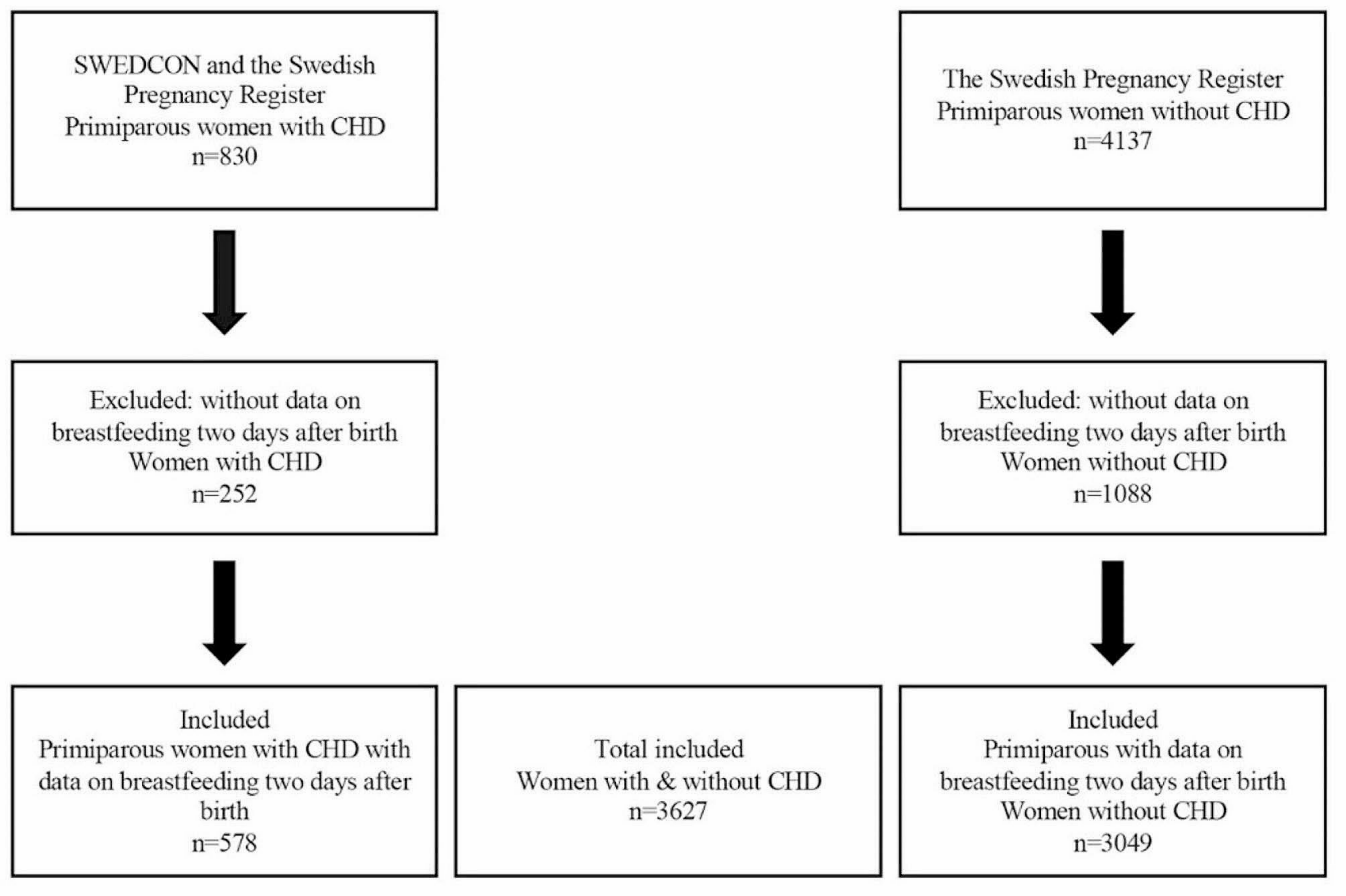
Flow chart of included and excluded women with and without congenital heart disease

The two registers were merged, and women with CHD who matched the inclusion criteria were selected: (i) having been diagnosed with CHD with at least one visit to an adult CHD clinic after the age of 18 years, (ii) being primiparous women who had given birth after 22 + 0 gestational weeks, and (iii) having data on breastfeeding two days after birth. These were matched approximately 1:5 by residential area and year of birth to women without CHD. The final sample comprised 578 women with CHD (Table 1) and 3049 women without CHD (see Fig. 1).

**Table 1:** Overview of women’s diagnoses of congenital heart disease

Diagnoses	n (%)
Ventricular septal defect	182 (31.7)
Arterial septal defect	111 (19.4)
Aortic valve disease	67 (11.6)
Pulmonary valve disease	59 (10.2)
Coarctation of the aorta	42 (7.3)
Tetralogy of Fallot	20 (3.5)
Marfan syndrome	12 (2.1)
Atrioventricular septal defect	8 (1.4)
TGA (arterial switch)	8 (1.4)
Ebstein anomaly	5 (0.9)
Pulmonary atresia with ventricular septal defect	5 (0.9)
TGA (atrial switch)	4 (0.7)
Truncus arteriosus	2 (0.3)
Double outlet right ventricle	1 (0.2)
Fontan / TCPC	1 (0.2)
Miscellaneous	50 (8.7)

*CHD* Congenital heart disease, *n* Number, *TCPC* Total cavo-pulmonary connection, *TGA* Transposition of the great arteries

### Outcome measures and analysed factors

The register splits the variable breastfeeding into exclusive, partial, or non-breastfeeding. Exclusive breastfeeding is when the baby receives only the mother’s milk from the breast or bottle. Partial breastfeeding is when the child regularly receives donated breast milk or formula in combination with breastfeeding. Breastfeeding was dichotomised into breastfeeding (both exclusive and partial) and non-breastfeeding. The analysis included factors related to the heart disease (complexity of the heart disease, NYHA class, use of cardiovascular medication, and symptoms related to the heart disease) where the complexities of lesions were classified according to European Society of Cardiology guidelines [24], comorbidities, obstetric complications, and sociodemographic characteristics. Comorbidities among included women were BMI ≥ 30, hypertension prior to pregnancy, diabetes mellitus, hypothyroidism, and self-reported history of psychiatric illness, current or previously. Obstetric complications were defined as pre-eclampsia, preterm birth (represented in this study, children born between gestational weeks 31 and 36), induced labour, vacuum extraction / forceps, Caesarean section, major *postpartum* haemorrhage > 1000 ml [25], perineal trauma III − IV, and baby small for gestational age (SGA) based on Marsál’s curve [26]. Sociodemographics included age, civil status, level of education, and tobacco use.

### Statistical analysis

Statistical analyses were conducted using version 28 of the IBM SPSS Statistics software package (IBM Corp., Armonk, NY, USA). All data were assessed for normality. Differences in means and ratios were tested using Student’s *t*-test, and Chi^2^ − test. Univariable logistic regression analyses were used to test whether non-breastfeeding at two days and four weeks after birth was associated with factors related to the heart disease, comorbidities, obstetric complications, and sociodemographic characteristics in all women and women with CHD separately. Independent factors that showed association with non-breastfeeding were further tested for collinearity using Spearman correlation. A correlation was seen between induced labour and Caesarean section, and between Caesarean section and premature birth. Due to this, Caesarean section was omitted from further analyses. Furthermore, missing data in the variables “NYHA” and “cardiovascular medication” resulted in a reduced total number in the multivariable analysis. Missing data was handled as an own category in these variables. In doing so, cases with missing data did not differ from the reference categories (i.e., “NYHA class I” and “no cardiovascular medication”) in the univariable regressions and were therefore merged with the reference category (NYHA 1 + missing data, no cardiovascular medication + missing data) in the following analyses. Finally, the remaining factors with a *p*-value < 0.1 in the univariable analyses were included in multivariable models and analysed in a stepwise backward elimination manner. The results from logistic regression are presented as odds ratios with 95% confidence intervals (95% CI), and the null hypothesis was rejected on *p*-values < 0.05.

## Results

Women with CHD were more likely to suffer from obstetric complications during pregnancy and childbirth, and self-reported history of psychiatric illness was reported more frequently. The level of education was slightly higher in women with CHD compared to women without CHD. An overview of the women’s characteristics is presented in (Table 2). Women with CHD had a lower breastfeeding rate two days after birth compared to women without CHD (94% vs. 97%, *p* = 0.001). Four weeks after birth, the breastfeeding rate in women with CHD was still lower compared to women without CHD (84% vs. 89% *p* = 0.006) (Table 3). Analyses on breastfeeding were performed on women who gave birth at full-term. The differences in breastfeeding were lower among women with CHD compared to women without CHD two days (95% vs. 97%, *p* = 0.028) and four weeks after birth (86% vs. 89%, *p* = 0.04). Univariable and multivariable analyses, including all women, women with and without CHD, were carried out to explore whether being diagnosed with CHD was associated with non-breastfeeding. The models showed that being diagnosed with CHD was associated with non-breastfeeding both two days (OR 1.8; 95% CI 1.2, 2.7) and four weeks after birth (OR 1.5; 95% CI 1.1, 2.0).

**Table 2:** Background characteristics of women with and without congenital heart disease (CHD)

Variable	All women n (%) / mean (SD)	Women with CHD *n* = 578 (100%)	Women without CHD *n* = 3049 (100%)	*P*-value
**Age in years**				
Mean (SD)	28.54 (4.6)	28.85 (4.7)	28.47 (4.6)	
Min − max	18 − 44	19 − 44	18 − 44	
**Sociodemographic factors**				
Living alone	79 (2.3)	12 (2.2)	67 (2.3)	0.86¹
≤ 12 years of education	1714 (51.6)	262 (49.3)	1452 (52.0)	0.26
Tobacco use in early pregnancy	168 (4.9)	19 (3.5)	149 (5.2)	0.09^1^
**Comorbidities**				
BMI ≥ 30	369 (10.8)	61 (11.2)	308 (10.8)	0.75
Hypertension before pregnancy	12 (0.3)	3 (0.5)	9 (0.3)	0.38¹
Diabetes mellitus	18 (0.5)	3 (0.5)	15 (0.5)	0.92¹
Hypothyroidism	141 (3.9)	26 (4.5)	115 (3.8)	0.41¹
Self-reported history of psychiatric illness	612 (17.5)	131 (23.7)	481 (16.3)	**< 0.001**
**Obstetric complications**				
Pre-eclampsia	137 (3.8)	30 (5.2)	107 (3.5)	0.052¹
Induced labour	773 (21.3)	146 (25.3)	627 (20.6)	**0.011**
Epidural during labour	109 (9.2)	19 (9.8)	90 (9,1)	0.74¹
Vacuum extraction/forceps	358 (9.9)	54 (9.3)	304 (10.0)	0.64
Caesarean section	595 (16.4)	133 (23.0)	595 (16.4)	**< 0.001**
Preterm birth	127 (3.5)	24 (4.2)	103 (3.4)	0.35¹
SGA	147 (4.1)	29 (5.0)	118 (3.9)	0.12^1^
Major postpartum haemorrhage > 1000 mL	332 (9.2)	63 (10.9)	269 (8.8)	0.11
**Factors related to the heart disease**				
Complexity of heart lesion	578 (100)			
Mild		414 (71.5)		
Moderate		151 (26.1)		
Severe		13 (2.2)		
NYHA class	261 (45.2)			
I		217 (94.3)		
II		12 (5.2)		
III		1 (0.4)		
Symptomatic heart disease		36 (11.4)		
Cardiovascular medication		37 (11.9)		

*BMI* Body mass index, *CHD* Congenital heart disease, *n* Number, *NYHA* New York Heart Association, *SD* Standard deviation, *SGA* Small for gestational age

Chi² test unless otherwise indicated: ¹Fisher’s exact test; Diabetes mellitus includes diabetes 1 and 2; Continuous variable: age; Variables with missing data > 10%: epidural *n* = 118, (32.6%), NYHA class *n* = 261, (51.2%), symptomatic heart disease *n* = 316, (54.7%), Cardiovascular medication *n* = 310, (53.6%); Bold highlighting denotes *p*-value < 0.05

**Table 3:** Breastfeeding in women with and without congenital heart disease (CHD)

Variable	Women without CHD, n (%)	Women with CHD, n (%)	p-value	Mild complexity n (%)	Moderate/severe complexity n (%)	*P*-value
**Breastfeeding two days after birth**	3049 (100)	578 (100)		414 (100)	164 (100)	
None	104 (3)	36 (6)		22 (5)	14 (9)	
Exclusive or partial	2945 (97)	542 (94)	**0.001**	392 (95)	150 (92)	0.15
**Breastfeeding four weeks after birth**	2186 (72)	405 (70)		290 (71)	115 (70)	
None	240 (11)	64 (16)		45 (16)	19 (17)	
Exclusive or partial	1946 (89)	341 (84)	**0.006**	245 (85)	96 (84)	0.80

*CHD* congenital heart disease

Chi² test and descriptive statistics: n, number; Bold highlighting denotes *p*-value < 0.05

### Factors associated with non-breastfeeding in women with CHD

Univariable logistic regression analyses showed that among women with CHD, non-breastfeeding two days after birth was associated with BMI ≥ 30 (OR 4.0; 95% CI 1.8, 8.7), self-reported history of psychiatric illness (OR 2.8; 95% CI 1.4, 5.6), preterm birth (OR 7.5; 95% CI 2.9, 19.4), SGA (OR 4.5; 95% CI 1.7, 11.9), and NYHA class II − III (with NYHA class I as reference) (OR 5.3; 95% CI 1.3, 18.4). The multivariable model showed that BMI ≥ 30 (OR 3.1; 95% CI 1.4, 7,3), self-reported history of psychiatric illness (OR 2.4; 95% CI 1.2, 5.1), and preterm birth (OR 6.4; 95% CI 2.1, 19.0), SGA (OR 4.2; 95% CI 1.4, 12.2), and NYHA class II-III (OR 6.0; 95% CI 1.4, 26.7) were associated with non-breastfeeding two days after birth (Table 4).

**Table 4:** Logistic regression in women with CHD, dependent variable: non-breastfeeding two days after birth

Variable	Univariable logistic regression				Multivariable logistic regression							
					Initial model, including *n* = 492 (85%)				Final model, including *n* = 531 (92%)			
	Wald	OR	95% CI	*p*-value	Wald	OR	95% CI	*p*-value	Wald	OR	95% CI	*p*-value
Higher age	0.1	1.0	0.9, 1.1	0.82								
≤12 years of education	2.8	1.9	0.9, 3.9	0.094	0.6	1.4	0.6, 3.1	0.43				
Living alone	0.1	1.4	0.2, 11.2	0.75								
Tobacco use in early pregnancy	0.1	0.8	0.1, 6.0	0.81								
BMI ≥ 30	12.7	4.0	1.8, 8.7	**< 0.001**	5.0	2.8	1.1, 7.1	0.026	7.2	3.1	1.4, 7.3	0.008
Self-reported history of psychiatric illness	8.6	2.8	1.4, 5.6	**0.003**	4.9	2.4	1.1, 5.3	0.027	5.5	2.4	1.2, 5.1	0.019
Hypertension prior to pregnancy	0.0	0.0	0.0	0.99								
Hypothyroidism	1.3	2.1	0.6, 7.2	0.26								
Pre-eclampsia	0.8	1.7	0.5, 6.0	0.39								
Induced labour	0.0	1.0	0.5, 2.1	0.97								
Vacuum extraction/forceps	0.6	0.6	0.1, 2.4	0.427								
Caesarean section	0.1	1.1	0.5, 2.5	0.77								
Postpartum haemorrhage > 1000mL	0.3	0.7	0.2, 2.5	0.61								
Perineal trauma III & IV	0.0	0.8	0.1, 6.4	0.86								
Preterm birth	17.0	7.5	2.9, 19.4	**< 0.001**	8.7	5.7	1.8, 18.3	0.003	10.9	6.4	2.1, 19.0	**< 0.001**
SGA	9.3	4.5	1.7, 11.9	**0.002**	7.9	4.7	1.6, 14.0	0.005	6.8	4.2	1.4, 12.2	0.009
Moderate & severe complexity	2.1	1.7	0.8, 3.3	0.15								
NYHA class II-III	5.1	5.1	1.2, 21.1	**0.024**	5.5	5.9	1.4, 26.0	0.019	5.6	6.0	1.4, 26.7	0.019
Symptomatic heart disease	0.1	0.8	0.2, 3.4	0.73								
Cardiovascular medication	0.2	1.3	0.4, 4.8	0.66								

*BMI* Body mass index, *CHD* Congenital heart disease, *CI* Confidence interval, *n* Number, *NYHA* New York Heart Association, *OR* Odds ratio, *SD* Standard deviation, *SGA* Small for gestational age

Univariable and multivariable regression analysis including variables with *p*-value < 0.1 from univariable logistic regression analysis; Continuous variable: higher age; Nagelkerke R square 0.18; Bold highlighting denotes *p*-value < 0.05

Four weeks after birth, the univariable logistic regression analyses showed that non-breastfeeding for women with CHD was associated with ≤ 12 years of education (OR 1.9; 95% CI 1.1, 3.3), BMI ≥ 30 (OR 4.4; 95% CI 2.2, 8.8), self-reported history of psychiatric illness (OR 2.2; 95% CI 1.2, 3.9), induced labour (OR 1.8; 95% CI 1.0, 3.2), Caesarean section (OR 2.7; 95% CI 1.5, 4.7), preterm birth (OR 6.8; 95% CI 2.4, 19.5), and treatment with cardiovascular medication (OR 2.8; 95% CI 1.1, 7.4). In the multivariable model, BMI ≥ 30 (OR 4.3; 95% CI 2.1, 9.0), self-reported history of psychiatric illness (OR 2.2; 95% CI 1.2, 4.2), and preterm birth (OR 8.9; 95% CI 2.8, 27.9) were associated with non-breastfeeding four weeks after birth (Table 5).

**Table 5:** Logistic regression in women with CHD, dependent variable: non-breastfeeding four weeks after birth

Variable	Univariable logistic regression				Multivariable logistic regression							
					Initial model, including *n* = 376 (65%)				Final model, including *n* = 378 (65%)			
	Wald	OR	95% CI	*p*-value	Wald	OR	95% CI	*P*-value	Wald	OR	95% CI	*P*-value
Higher age	0.2	1.0	0.9, 1.1	0.63								
≤12 years of education	2.2	1.9	1.1, 3.3	**0.02**	1.6	1.5	0.8, 2.8	0.21				
Living alone	0.3	1.8	0.2, 17.7	0.61								
Tobacco use in early pregnancy	2.1	2.5	0.7, 8.3	0.144								
BMI ≥ 30	16.7	4.4	2.2, 8.8	**< 0.001**	12.2	3.9	1.8, 8.2	< 0.001	15.1	4.3	2.1, 9.0	< 0.001
Self-reported history of psychiatric illness	6.8	2.2	1.2, 3.9	**0.009**	6.1	2.3	1.2, 4.3	0.013	6.1	2.2	1.2, 4.2	**0.014**
Hypertension prior to pregnancy	0.0	0.0	0.0	0.99								
Hypothyroidism	0.0	1.1	0.3, 3.8	0.92								
Pre-eclampsia	0.1	1.2	0.4, 3.7	0.75								
Induced labour	4.2	1.8	1.0, 3.2	**0.041**	3.0	1.8	0.9, 3.3	0.083				
Vacuum extraction/forceps	0.4	0.7	0.3, 1.7	0.55								
Caesarean section	11.4	2.7	1.5, 4.7	**< 0.001**								
Major postpartum haemorrhage > 1000mL	2.6	1.9	0.9, 4.1	0.106								
Perineal trauma III & IV	0.4	0.5	0.1, 4.2	0.54								
Preterm birth	12.8	6.8	2.4, 19.5	**< 0.001**	12.4	8.0	2.6, 27.4	< 0.001	13.9	8.9	2.8, 27.9	**< 0.001**
SGA	1.3	1.8	0.7, 5.3	0.25								
Moderate & severe complexity	0.1	1.1	0.6, 1.9	0.80								
NYHA class II-III	2.6	3.3	0.8, 14.2	0.108								
Symptomatic heart disease	0.2	1.3	0.4, 4.1	0.67								
Cardiovascular medication	2.9	2.2	0.9, 5.5	0.091	3.9	3.0	1.0, 8.6	0.048				

*BMI* Body mass index, *CHD* Congenital heart disease, *CI* Confidence interval, *n* Number, *NYHA* New York Heart Association, *OR* Odds ratio, *SD* Standard deviation, *SGA* Small for gestational age

Univariable and multivariable regression analysis including variables with *p*-value < 0.1 from univariable logistic regression analysis; Continuous variable: higher age; Nagelkerke R Square 0.15; Bold highlighting denotes *p*-value < 0.05

Analyses were conducted on cases without breastfeeding data showing this group to be more affected by the heart disease, with more women having symptoms from the heart disease (11.4% vs. 25.9%, *p* = < 0.001), more women having an NYHA class II-III (5.7% vs. 13.8%, *p* = 0.007), and more women having moderate and severe complexity (22.5% vs. 30.2%, *p* = 0.019). They were also more often affected by obstetric complications such as Caesarean Sect. (23% vs. 32.1%, *p* = 0.006) and preterm birth (4.2% vs. 27.7%, *p* = 0.001).

## Discussion

This register study examined breastfeeding rates two days and four weeks after birth in 578 women with CHD compared to 3049 women without CHD, as well as hindering factors for non-breastfeeding in women with CHD. The results show that women with CHD had slightly lower breastfeeding rates than women without CHD (two days after birth: 94% vs. 97%; and four weeks after birth: 84% vs. 89%), although both groups showed a relatively high level of breastfeeding compared to the general population of women in other European countries [10]. One explanation for the high level of breastfeeding in the present study might be the long paid parental leave in Sweden [10]; another might be the society’s high level of breastfeeding acceptance, which significantly affects women’s breastfeeding success [18].

Since sociodemographic characteristics, health history, and obstetric complications were included, this study was able to show associations of non-breastfeeding with both social factors and obstetric complications, and a few heart-related factors in women with CHD. However, the heart-related factor NYHA class II-III was only associated with non-breastfeeding two days after birth and not four weeks after birth. One reason for this could be that included women were relatively young with a mean age at 29 years, and not yet exposed to comorbidities in their heart disease, such as cardiovascular medication, symptoms from their heart disease or a higher NYHA class. Furthermore, women with severe conditions may have been advised against pregnancy [15]. For women with CHD, preterm birth showed the highest OR for non-breastfeeding, both two days (OR 6.4) and four weeks after birth (OR 8.9), and this has also been shown as a breastfeeding reducing factor in the general population [27]. However, previous studies have reported preterm birth rates of up to 16% among women with CHD [16], and so from an international view, the preterm birth rate (4.2%) in the present sample can be considered low. This may be one reason why breastfeeding rates did not differ substantially in the current sample [27]. Other factors associated with non-breastfeeding were BMI ≥ 30, and self-reported history of psychiatric illness, which again are factors known to impact breastfeeding among women in general [17, 28, 29].

In line with our finding of lower breastfeeding rates among women with CHD than women without CHD, previous research describes women with chronic illness as more likely than healthy women to cease exclusive breastfeeding [30]. A possible explanation for the lower breastfeeding rates among women with CHD in the current study could be that there is less consideration of whether the infant should receive breast milk substitutes. The new mother, her family, and her healthcare professionals can all be influenced by breastfeeding expectations [18], and as the woman has a chronic disease people around her may think that they should not stress her about breastfeeding. However, the initiation of breastmilk substitutes (e.g., as a complement) is a known risk factor for breastfeeding cessation [29]. Moreover, studies have found that women who wish to breastfeed but fail to do so express dissatisfaction, personal stigma, and even a sense of failure [31, 32]. In addition to this, women with CHD have an increased risk of emotional distress during their postpartum period [33], and unsuccessful breastfeeding may be perceived as an additional stressor for these women.

These novel results highlight that CHD combined with preterm birth, BMI ≥ 30, or psychiatric illness history may add a further complex dimension to the situation. As preterm birth and a history of psychiatric illness are more common among women with CHD than among women without CHD [16], one could say CHD may affect breastfeeding indirectly through these factors. However, women experiencing preterm birth, BMI ≥ 30, or having a psychiatric illness history may be an extra-vulnerable group in terms of successful breastfeeding. Whether or not a woman intends to breastfeed exclusively, our results indicate that formula should be given solely on strict medical indication in order to avoid jeopardising lactation onset. Our data show that for a high proportion of women with CHD breastfeed, however, CHD is still a factor associated with non-breastfeeding. Therefore, healthcare personnel should pay attention to breastfeeding even if a high proportion of women with CHD breastfeed.

A strength of the study is the large sample, with comparable previous breastfeeding experience of participants, as only primiparous women were included. However, as in all register studies, the data were limited to those included in the register. In cases of non-significant results in some comparisons, the possibility of being underpowered cannot be excluded. As complete data on the exact method of cardiac intervention in all lesions were not available, we were unable to investigate whether a median sternotomy affected the breastfeeding rate. The results also showed a higher proportion of women without data on breastfeeding giving premature birth compared to those with data on breastfeeding. Since premature babies are cared for at a higher level of care, and in many cases with separate records, breastfeeding data are not automatically transferred into the Swedish Pregnancy Register. As premature birth is associated with non-breastfeeding, our data may underestimate the prevalence of non-breastfeeding.

## Conclusion

The study shows that most women with CHD breastfeed, however, at a slightly lower proportion compared to women without CHD. In addition, factors related to the heart disease were not associated with non-breastfeeding four weeks after birth. Since preterm birth, BMI ≥ 30, and psychiatric illness are associated with non-breastfeeding, healthcare professionals should offer greater breastfeeding support to women with CHD having these conditions.

## Data Availability

No datasets were generated or analysed during the current study.

## Abbreviations

ACHD: Adult congenital heart disease
BMI: Body mass index
CHD: Congenital heart disease
CI: Confidence interval
NYHA: New York Health Association
OR: Odds ratio
SGA: Small for gestational age
SWEDCON: Swedish Congenital Heart Disease Register
WHO: World Health Organization
